# Traumatic Tetanus

**Published:** 1882-11

**Authors:** Daniel R. Brower

**Affiliations:** Professor of Mental and Nervous Diseases, Woman’s Medical College, Physician of Nervous Diseases at St. Joseph Hospital, and Consulting Physician to Nervous Diseases at Woman’s Hospital State of Illinois


CHICAGO MEDICAL

JOURNAL & EXAMINER.

Vol. XLV.—NOVEMBER, 1882.—No. 5.

### Original Communications.

### Article I.

Traumatic Tetanus. Recovery under Quinine and Alcohol. Sphæro Bacteria Present in the Blood. By Daniel R. Brower, m.d., Professor of Mental and Nervous Diseases, Woman's Medical College, Physician of Nervous Diseases at St. Joseph Hospital, and Consulting Physician to Nervous Diseases at Woman's Hospital State of Illinois.

P. M., æt. fourteen; German; light complexion; athletic; muscles well developed, received a "toy pistol" wound in right natis, on July 4, 1882. A comrade placed the muzzle of the pistol against his clothing, and an explosion accidentally occurred. The cartridge used in the toy pistol contains no ball, but is charged with fulminate of mercury, (as determined by analysis of Prof. W. S. Haines) in unusually large proportions, gunpowder, and a wad made of heavy pasteboard, the whole enclosed in a copper case. The large proportion of the fulminate of mercury causes these pistols to make a loud explosion, and gives great propulsive power to the wad. The great laceration of tissue these blank cartridges will produce, is surprising. Indeed, it was difficult, in this case, and more so in another I was called upon to examine, to convince one's self that the cartridge contained no ball.

In the case of P. M., there must have been carried into the wound, a portion of his coat, pants, shirt and drawers. The boy suffered so little inconvenience from the wound, that no physician was called at the time of the injury. The boy is a German of a lymphatic temperament, with nervous system quite free from irritability. The father died of an acute disease, aged fifty; his mother is living in excellent health, and is possessed of the same temperament as himself; his grandparents on both sides lived to old age, and no history of nervous disease was found in the family.

On the morning of the 16th of July, I was called to see him, and found him suffering from trismus, that had commenced the night before, and complaining of an acute pain in the præcordial region, with great difficulty in breathing, restlessness, a quick pulse, a temperature one and one-half degrees above normal, and bowels constipated. I found the wound in the integument, in the area of distribution of the ilio-hypogastric nerve, and on pressure quite offensive pus came from the bottom of it. The probe was then introduced, and the wound found to be about one inch and a half deep, flask-shaped, at least one inch in the largest diameter. I enlarged the wound, cleansed it with a solution of carbolic acid, and packed it with iodoform. The integument supplied by the hypogastric area of the ilio-hypogastric nerve was exceedingly sensitive to the touch, and the abdominal muscles were very rigid. I administered at once ten grains of calomel, and ordered a mixture containing twenty grains of bromide of potassium and twenty grains of chloral hydrate, to the dose, to be taken, and repeated in two hours if the patient was awake, and the dose to be repeated at that interval, until sleep resulted. The room he occupied was thoroughly darkened, and every one except his nurse excluded. He was ordered to be fed on milk punch, eggs, and beef soup.

I saw the patient at nine P. M. He was then sleeping; he had taken four doses of the bromide and chloral mixture. I ordered him to be liberally fed, on awaking; the room kept darkened; every one but his attendant excluded; and mixture continued as before. His pulse was 88; temperature of axilla 100; skin in a profuse perspiration. The urine he had passed was high-colored and scanty, specific gravity 1023, and strongly acid; after standing a deposit fell, consisting chiefly of the phosphates. The bowels still constipated.

The morning of the 17th found him awake; the præcordial pain less severe; trismus unchanged; had severely bitten his tongue, and had had a severe attack of opisthotonus during the night. No movement of the bowels having occurred, ordered an enema of castor-oil and soap-suds, the liberal use of milk punch, beef soup, and eggs, and the bromide and chloral mixture continued every three hours. In the evening, the patient's condition was unchanged; he had one attack of opisthotonus, much more severe than first one. Treatment continued, with addition of one ounce of sulphate of magnesia, as no movement of bowels had taken place.

The morning of the 18th, I had Dr. R. G. Bogue see the case in consultation. At his suggestion, a drainage tube was introduced into the wound, to make sure of the free discharge of the pus, and physostigma added to the treatment. I had in my pocket at the time, an aqueous extract of physostigma that had been prepared from a reliable sample by E. H. Sargent & Co., and one half of a grain was administered at once, by the mouth, and the dose ordered repeated every two hours, and the bromide and chloral mixture continued as before.

On the evening of the 18th, found the bowels had moved; and the patient more comfortable.

The morning of the 19th, found the pulse 90 and feeble; temperature 99°; body covered with perspiration; pupils contracted; had had during the night several severe attacks of opisthotonus, and had bitten the tongue badly; trismus unchanged. Treatment continued.

On the morning of the 20th, found the patient no better; attacks of opisthotonus severe and frequent; strength of patient much exhausted; complains very much of the chloral and bromide mixture; the tongue being swollen and tender, the chloral doubtless irritating it. He was thoroughly under the influence of the mixture; the face contained many lesions of bromide acne, and the mind was much disturbed; insisted upon it that his nurses were trying to kill him. His limbs were now in a constant state of rigidity; he complained of no pain, and slept most of the time. The chloral was omitted from the treatment.

On the 21st of July, found his condition unchanged, with the exception of more exhaustion. His pulse was feeble; temperature 100°; body bathed in profuse perspiration; mind much disturbed by delusions. The muscles of lower extremities and of the abdomen are quite rigid; has had attacks of opisthotonus about every hour of the night. He had great difficulty in swallowing, and was suffering great pain in the præcordial region and in both groins. One-fourth of a grain of morphia sulphate was given at once, and ordered to be repeated, if necessary, in two hours. The other treatment continued.

On the 22d of July, I took Dr. Lester Curtis to see the patient, to have him make a microscopic examination of the blood. The patient was much worse; paroxysms more violent, and suffering more acute. The examination of the blood revealed great quantities of sphæro-bacteria. We estimated sixty to the drop. The discovery of these micrococci caused us to immediately change treatment. Regarding alcohol and quinine as the antiseptics *par excellence*, and believing them to be the only ones with which the body can be surcharged without danger to the tissues, we began at once their administration. We gave him four ounces of brandy, and ordered him to receive as much as it was possible to administer, and every fourth hour, in addition, five grains of quinine. We saw him about twelve hours afterward, and found a marked improvement in his condition. He was more comfortable; the spasms had diminished in force and frequency; his pulse was 80 and stronger; temperature 99°; perspiration markedly less. This treatment by brandy, quinine and morphia, together with as much nourishment as possible, was continued, and the improvement went on without interruption. The brandy was given in as large quantities as he could be induced to swallow, the quinine continued at regular intervals, and morphia given as necessary to allay pain. A trial was made on several occasions of the nitrite of amyl, and for a time we thought it diminished the force of the attacks of opisthotonus. It was given just before the onset of a paroxysm, but it was very disagreeable, and soon lost its seeming efficiency.

The blood was examined again by Drs. Curtis and I. N. Danforth, and the micrococci found as before. An effort was then made to test these revelations by other cases of tetanus, but after a careful inquiry, no other one could be found in the city, except that of a horse, whose blood, upon examination, showed the same kind of bacteria in abundance. A case of reported tetanus in the Cook County Hospital, in the homœopathic department, was examined by Dr. Curtis and myself, and no bacteria found in the blood; but we both regarded the case as one of hysteria, and not of tetanus. We thought our experience had established a bacterial foundation for the pathology of tetanus, but, at the suggestion of Dr. Curtis (who, by the way, is a most conscientious, painstaking and conservative observer, with a large experience in the microscopy of blood), we examined the blood of the mother and sister of my patient, and, to my great surprise, found the same sphæro-bacteria here in abundance. Three weeks later, when my patient was well, with the exception of a great degree of stiffness of the injured limb and some debility, his blood, and that of the mother and sister, were again examined, and bacteria found as before. The mother and daughter seemed in excellent health. The strain upon them had been great. The mother had an infant six months old she was nursing, a picture of perfect health, and, in addition to the care and attention the patient constantly required, night and day—which demand was almost entirely met by herself and daughter, she attended to other necessary household duties ; seeming to demonstrate the fact that sphæro-bacteria are not incompatible with great capacity for endurance and the development of robust infants. And, moreover, that sphæro-bacteria may be present in the blood of a patient suffering from tetanus; may be present during every stage of the disease, from its accession to its subsidence, without in any way influencing unfavorably the progress of the disease toward recovery, or modifying in the slightest degree its characteristic manifestations. The prevalence of bacteria in this family led us to investigate more carefully their surroundings. They live in the principal story of a house with windows opening south, on a vacant lot, much below the level of the street, and in this vacant lot there was a large pool of stagnant water. Some of this water was examined by Dr. Curtis, and the same kind of bacteria there found in abundance.

These developments suggest the possibility that the micrococci found in cerebral spinal meningitis by M. Ernest Grandier (*Revue Médical*, June 3, 1882), and the bacteria found by Baumgarten in hydrophobia, and by Keating in malignant measles, may have a similar origin, and have no more relation to the etiology of the diseases they describe, than the bacteria in the blood of my patient, or in the blood of the horse, had to tetanus; and, indeed, that bacteria generally may be purely accidental, even in phthisis pulmonalis, notwithstanding the brilliant discoveries of Koch, and the reported cure of consumption by continuous inhalations of antiseptics. The carbolic spray of Lister is no longer fashionable, and bacteria to-day are not held responsible for all the failures of surgery, especially as they have been found in wounds where every Listerian precaution was taken, so that Mr. Lister himself acknowledges that bacteria can work no harm to the body while the vital action is maintained in its integrity.

We call the attention of the readers of this article to the admirable paper of Dr. Lester Curtis that accompanies it. In this paper, the doctor simply describes what he saw, without calling the bodies by their specific names, but he has no doubt about their being bacteria. We have together examined them in comparison with the sphæro-bacteria from a pyæmic abscess, and found them precisely the same in their reactions to light, in their movements, and their properties generally. The slides were carefully examined by Drs. Boerne Bettman, and Roswell Park, of this city, who are fresh from the study of bacteria in the laboratories of Germany, and by Dr. I. N. Danforth, one of the leading pathologists and microscopists of the Northwest; and they agree with Dr. Curtis that the bodies found are bacteria.

The bacterial origin of tetanus having been disposed of, we are forced back upon the nervous system to find the morbid anatomy of its pathological manifestations. Here we are surrounded by uncertainty, for no one lesion has been described which may not be found in cases of ordinary paralysis, progressive muscular atrophy, locomotor ataxia, and kindred diseases, that manifest no single symptom of tetanus; and furthermore, notwithstanding the most careful observation of the cerebral nervous system by competent pathologists, cases have occurred in which no lesion whatever has been discovered; so that we are forced to the conclusion that the lesions described are probably the effects, rather than the cause, of the phenomena of tetanus.

The lesion most frequently found is vascular engorgement of the spinal cord, and subsequent exudations and disintegrations. Dr. E. L. Fox (Path. Anatomy of Nervous Centers, page 355) gives the result of careful microscopic examination of five cases. In the first, the cord was perfectly healthy, the only abnormality found being engorgement of the vessels of the spinal pia mater. In the second, the cord was universally softened in its whole extent and thickness. In the third, the spinal cord and membranes were healthy down to a point corresponding to the tenth or eleventh dorsal vertebra; here there was effusion of blood outside the spinal dura mater, and for a half inch softening of cord. In the fourth case, the softening was limited to the posterior columns of the cord, except at the very top, where one inch of cord was involved in the necrosis. Demme and Rokitansky describe lesions similar to those of sclerosis, disseminated throughout the entire spinal cord. Wiegand recognized analogous appearances, in particular, hypertrophy of the neuroglia elements which are found in the gray commissure surrounding the central canal.

Cornil and Ranvier, in their examination of analogous cases, found a normal condition in this location.

Dr. Joseph Ross (Diseases of Nervous System, Vol. I, page 850) found marked softening in the lumbar region, and infiltration of the cord generally with leucocytes; but the most interesting changes were observed in the ganglion cells of the anterior horns. The cells of the median group and the marginal cells of the other groups, under a low power, seemed to have disappeared, but under a higher power they could be seen shrunken in their cavities. The cells of the antero-lateral, and a few of those of the postero-lateral groups, were of normal size. The inferior portion of the nucleus of the hypoglossal, spinal accessory and pneumogastric all suffered. The root of the fifth nerve proceeding from the cerebellum, showed large vessels distended with red blood-corpuscles, and the whole surrounding tissue was densely infiltrated with leucocytes. The corpus dentatum of the cerebellum, and white substance adjacent, was also densely infiltrated with leucocytes, and intersected with distended blood-vessels. The cells of Purkinje were also surrounded with leucocytes. The accessory nuclei were found nearly destroyed by the same degeneration.

Dr. William Aitken (Science and Practice of Medicine) says: "My own observations on the specific gravity of portions of the cord in tetanus, lead me to the belief that the blood vessels, and thus vascularity rather than nerve tissue lesion, have much to do with the phenomena." He found a marked increase in the specific gravity of the cord in those parts connected with the nerves going to and proceeding from the wound, as compared with other portions of the cord.

To sum up these central changes, we find the evidence of disturbed vascularity, probably hyperæmia; and that this disturbance of nutrition sooner or later produces degeneration. It is not surprising that there should be found in other diseases more or less dependent upon disturbances in vascularity, the same pathological changes that we have in tetanus. They are not the beginning of the disease. Just as the degenerations are secondary to the vaso-motor disturbances, so are the vaso-motor disturbances secondary to something else. We must look for this something in the condition of the peripheral nervous system, especially in the sensory end organs. These end organs are just as essential to the manifestations of nervous phenomena as the centers are, and we have no doubt that they are the origin of much that is obscure to day in neuro-pathology. Something of the important relations that we consider these organs have, may be seen by instituting a comparison between the ordinary manifestations of electricity and nerve force. Take the ordinary telephone, and we find the microphone to be its end organ; the battery is its central nervous system; the wires its peripheral nerves. Now, this telephone may be rendered useless by a section of its wires; by a failure of its battery; but just as much so by an injury to its microphone. In every case of traumatic tetanus where careful examination has been made of the nerves leading from the wound, there has been found evidence of congestion; of alteration of texture; of inflammation.

Dr. W. A. McDowell (*New Orleans Medical Journal*, March, 1846) has induced tetanus by the introduction of irritating bodies into the nerves. He introduced minute tack points into the musculo-spiral nerve of one dog, and into the ulnar nerve of another dog, passing the tacks completely through the nerves, burying both extremities within the theca. Tetanus developed in one in six days, and in the other in three days afterward.

Mr. Poland has shown (Guy's Hospital Reports, 3d Series, Vol. III,) that lacerated wounds are much more frequently attacked with tetanus than incised wounds; that the disease occurred only in one case out of 1,364 where the wound was made with a sharp knife, but it ensued in one out of 55 where the nerves were injured, as in an accident.

Baron Larrey, whose personal observation of the disease was exceedingly extensive, held the view that irritation is first set up in the wound and injured nerves, and recounts several cases in which the disease was either cured or greatly relieved by section of the nerves or amputation of the limb. He also records several cases in which recovery followed the application of the actual cautery to the wounded surface and to the extremities of the injured nerves.

Mr. Erichsen (Science and Practice of Surgery, page 578), who made numerous post-mortem examinations of traumatic tetanus, said that the only morbid condition that is constantly found, is a degree of inflammation of the nervous twig leading from and implicated in the wound, and the vascularity may be traced up the neurilemma, often for a considerable distance. He relates several instances in which recovery resulted from section of nerves, and advised, in those cases in which no special nerve appears to have been injured, Liston's recommendation cf making a V-shaped incision down to the bone above the part, so as to insulate it completely.

Langenbeck (Syd. Soc. Year-Book; p. 220, 1863) gives the record of three cases that sustain the view that tetanus is dependent on local irritation. In the first, the removal by an incision of a fragment of a needle was followed by an immediate subsidence of the symptoms and recovery of the patient. In the second, the removal of a ligature that had been tied *en masse* after castration, at once stopped all the symptoms. In the third case, the reduction of a fracture which was attended with great displacement, had the desired effect.

Pelletier, Froriep, Curling, Hasse and others, record numerous cases corroborative of the origin of tetanus in peripheral irritation. The fact of sudden atmospheric changes, as from warm to cold, increasing the tendency to tetanus, has been generally recognized. I can bear witness to this in three cases of traumatic tetanus that developed in my service in the U. S. Army, 1864. These wounded men were doing well, until a sudden change in the atmosphere occurred, the wind shifting suddenly in the night from south to east, and they occupying beds beside open north windows. They were almost immediately seized with trismus, and in two of the cases death speedily followed. This fact in the etiology of tetanus strengthens the position we are trying to maintain. The essential pathological factor in the impress of cold, is its effect upon the sensory end organs of the peripheral nerves. The chill experienced is the commotion set up in these organs and transmitted to the brain. The pains "all over;" the aching of the joints, limbs and back; and sense of tightness across the forehead, a part of the history of every "common cold," is but the expression of irritation, the result of disturbed nutrition in these same sensory end organs. We relieve this end organ depression, if we give promptly, before vaso-motor disturbances are established in the centers, stimulating doses of morphia, quinine and alcohol; remedies that have in small doses a stimulating effect, especially upon this important part of the nervous system.

If we have the sensory end organs already in a state of irritation, as the result of a lacerated wound or other cause, the impress of cold may be sufficient to set in motion the phenomena of tetanus. The irritation in the end organs and peripheral nerves is propagated to the delicate network of cells in the spinal system, and produces there an intense irritation that gives rise to hyperæmia, and the hyperæmia, on account of the excessive sensibility of these histiological elements, may of itself give rise to reflex spasms. The prompt disappearance of these congestions, before they have produced marked tissue changes, will explain the fact that some cases terminate favorably. The medulla oblongata is first to receive the impress of these irritations, not only because of the greater sensibility and more complex action of its nerve cells, but because of intimate connection with the sensory end organs. We know that when the functions of the medulla are depressed, as by lethal doses of opium and belladonna, we arouse the dormant powers by stimulants applied to the skin.

This intimate relation between the sensory end organs and the respiratory center, and the close anatomical and physiological connections between this center and the centers of the trigemini and facial nerves, will explain the tonic spasms of the face and jaw, the embarrassment of respiration and pain in the chest, with which the disease begins.

The area of diseased action extending in the medulla, and the nuclei of the hypoglossal, pneumogastric, glossopharyngeal and spinal accessory nerves, will occasion disorder of speech, deglutition, and still further embarrassment of respiration. The sweat center, and the convulsive center, participating in this disturbed nutrition, will give rise to characteristic phenomena.

The presence of sugar or of albumen, so often found in tetanus, is of interest in connection with this irritation of morbid action in the medulla.

We endeavored to ascertain why the "toy pistol" should have resulted in so many cases of tetanus, and made experiments with the fulminate of mercury upon one dog and three cats. It was our impression that this agent might be especially irritating to the nervous system because of the close analogy suggested by Prof. Haines, in composition, between fulminic acid and hydrocyanic acid.

In these experiments, lacerated wounds of the paws were made, and in these wounds the fulminate was placed in various quantities. We made in all nine lacerations, and used the fulminate in every proportion from a minute quantity up to the entire amount of the fulminate found in a cartridge, and in every instance the animals recovered without showing any perturbation of the nervous system.

The great number of cases of tetanus is to be accounted for by the great laceration of tissue produced by the excessive amount of propulsive force due to the large quantity of the fulminate in the cartridge, and to the fact that the great majority of the wounds were in the hand, that part of the body that is most richly endowed with sensory end organs.

The theory of the pathology of tetanus demands, in the matter of treatment, careful attention to the wound itself; removal of foreign bodies; dilatation of wound where secretions are retained, and the local use of anodynes. The anodyne *par excellence* for this purpose is iodoform. Not only is it a very powerful local anæsthetic, but it has positive antiseptic properties. The wound, after proper cleansing, should be well packed with this substance.

The nerve leading from the wound should, if possible, be resected before the disturbances in nutrition in the centers have produced degeneration. Its success depends upon its promptness.

In our opinion, nerve section is preferable to nerve-stretching, because of its certainty in cutting off communication. If the stretching is not severe enough to arrest the functional activity of the nerve, it can do no good.

Dr. W. J. Chandler has gathered together (*Medical Record*, September 9, 1882) the reports of fifty cases of nerve-stretching in traumatic tetanus, with nine cures and three reliefs. Since this compilation, Dr. E. W. Lee, of this city, has stretched the sciatic and crural, with immediate relief and subsequent recovery. Where the wound is so located that there is doubt about the peripheral connection, Liston's method, endorsed by Erichsen, of making a V-shaped incision about the part, so as to insulate it completely, may be advantageously followed.

The drugs in my judgment to be preferred are quinine, in full doses, alcohol and opium. Quinine, thus administered, diminishes the reflex activity of the spinal cord; it is a sedative to the circulation; it arrests the migration of leucocytes.

Mr. A. Poland (Guy's Hospital Rep., 3rd ser. vol. III) gives the record of twenty-five cases treated with quinine with seven recoveries. Dr. J. Jones, (*Medical and Surgical Review*) reports six cases treated with quinine, with one death. Dr. Hosmer A. Johnson successfully treated a case of tetanus following lacerated wound of thumb, with ten grain doses of quinine every four hours, and a case of trismus nascentium with one grain doses of quinine. Dr. Johnson was induced to use quinine in these cases because of a similarly successful experience of Dr. Herrick, of this city.

Alcohol in large doses is indicated, because it diminishes the reflex powers of the spinal cord, arrests to a certain extent tissue metamorphosis, produces sleep, reduces animal temperature, and acts as a nutrient.

Dr. Jones reports five cases of alcohol treatment, with one death.

Opium must be beneficial, because of its power of producing sleep and relieving pain, yet it must be used with caution, because of its primary stimulating effect upon the spinal cord, its tendencies to derange digestion and produce constipation.

The patient should be kept in a darkened room, free from all external irritation, and furnished with an abundance of nutritious food. The danger of death from asphyxia must be guarded against by the use, when necessary, of artificial respiration.

*Tabular Statement of Thirteen Fatal Cases of "Toy Pistol" Tetanus, in Chicago during July,* 1882—*Ten other Fatal Cases occured, but the Physicicians who treated them would not report them.*

| *No.* | *Age of Patient* | *Sex.* | SITUATION OF WOUND. | TETANUS DEVELOPED. | TREATMENT. |
|---|---|---|---|---|---|
| 1 | 9 | M. | In web bteween middle and ring finger. | After five days. | Chloral hydrate. Died on second day. |
| 2 | 14 | M. | Palmar surface of left thumb. | After four days. | Bromide potassium; gelseminum; curara; morphia; salicylic acid. Died on third day. |
| 3 | 8 | M. | Palm of leit hand between third and fourth metacarpal bones. | After eleven days. | Doctor was called when patient was dying. He had been treated with chloroform, cypropedium and valerian. |
| 4 | 9 | M. | Palmar surface of left hand. | After five days. | Hypodermics of morphia. Died on third day. |
| 5 | 9 | M. | Palmar surface of left hand. | After ten days. | Carbolic acid locally; chloral; bromide; nerve-stretching. Died on fourth day. |
| 6 | 13 | M. | Palmar surface of left hand. | After eight days. | Bromide ot potassium and chloral; ether spray to spine; chloroform; morphia. Died after four days*. |
| 7 | 15 | M. | Palmar surface of left hand. | After eight days. | Bromide potassium; nerve-stretching; hydrate chloral; morphia; calabar bean Died on third day. |
| 8 | — | M. | | Free open'g of wound. | No medicine taken. Died on second day. |
| 9 | 21 | M. | Just above right supra-orbital foramen. | After seven days. | Patient could not swallow. Gave 90 grs. bromide and 60 grs. chloral hydrate by enema. Died on second day. |
| 10 | 27 | M. | Palm of left hand near thumb. | After seven days. | Chloroform and hot baths. |
| 11 | 13 | M. | Palmar surface, first finger left hand. | After twelve days. | Carbolic lotions locally; chloral; bromide potassium and calabar bean. Died on eleventh day. |
| 12 | 7 | M. | Scrotum. | After five days. | Calabar bean and hot baths. Died on third day. |
| 13 | 9 | M. | Palm of right hand. | After eleven days. | Bromide of potassium; calabar bean; chloral hydrate; ice to spine; salicylate of sodium. Died on second day. |

* The doctor reports two cases in which the free use of opium and carbolic acid locally arrested a developing tetanus.

